# Paris Letters

**Published:** 1882-09

**Authors:** R. Tilley

**Affiliations:** Paris


### Foreign Correspondence.

ARTICLE VIII.

PARIS, JULY 22, 1882.

EDITORS MEDICAL JOURNAL AND EXAMINER:

Without pretending to give a complete account of any individual clinic here in Paris, I will endeavor to arrange a few items from my note-book, embracing general information, as well as particulars of clinics.

Foreign medical men are everywhere cordially received in Paris. All the hospitals and clinics are open to them free of charge, and on the presentation of professional cards special attention is invariably afforded. Even the histiological laboratories, with their advantages, are also at their service. If a stranger wishes to work in the histiological laboratory of the College of France, under Bauvier, he has only to present his card as physician, enter his name, and if all the tables are not occupied, he can begin work at once, provided he has with him his microscope. It is, however, absolutely essential to understand French. In the German and Austrian hospitals nearly every one speaks more or less English, but that is by no means the case in France. It must, however, be readily conceded that a person not understanding the language of the hospital is at a decided disadvantage.

One of the first places that attracts a medical man in Paris is the Hospital of Saltpetriere, the scene of the labors of Charcot. He finished, two weeks ago, his first course in the capacity of Professor of Nervous and Mental Diseases of the Faculty of Medicine. The clinic was given on Thursday and Sunday mornings, and lasted from half past nine to eleven o'clock; it was given in a large hall completely darkened, and lighted with gas.

Every possible convenience for screen projections and illustrations are provided, and dexterously manipulated by his numerous assistants. I had the pleasure of witnessing some of those results of pressure on the ovaries of which I had previously read, but never before seen illustrated with such marked results. I wish I could give to the readers of the JOURNAL some realization of the picture. One of the first patients presented was a young girl of charming appearance. The only visible sign of a departure from normal physiology was a persistent inward contraction of right foot. She was, however, wearing a ceinture which produced pressure in the region of the ovaries. The ceinture was removed, and immediately a violent fit of coughing was developed, which, even for the short time that it was exhibited, was positively painful to observe. The ceinture was re-applied, and the coughing ceased as by magic. Another patient was presented, with whom the removal of the ceinture was followed by the regular development of the various stages of epilepsy, exhibiting all the violence of agitation, frothing at the mouth, rapid, powerful muscular movements, followed by the most complete opisthotonus. The application of the ceinture cut short these paroxysms at any particular stage of their development with the most remarkable promptitude. Some half dozen patients were presented, illustrating in a similar way the same influence. In one case, when the removal of the ceinture was not followed immediately by an onset of the epileptic attack, the assistant gave a very slight but rapid tangential blow of the hand in the small of the back, and immediately the epileptic attack began, culminating in the cataleptic condition.

One case was exhibited of unusual interest, on account of its history. Becoming pregnant, it was found that the points on which pressure had to be exerted in order to relieve the attacks of epilepsy, gradually ascended as the pregnancy developed.

The ceintures which are used at the Saltpetriere are of various forms, in many respects similar to trusses. Although this particular branch of medicine bears no immediate relation to the branch to which I am giving special attention, as Messrs. E. H. Sargent & Co. authorized me to purchase for them anything which I should think of special use to the profession, an opportunity will be afforded of seeing the latest modifications of these ceintures in Chicago in a short space of time.

On another occasion a series of hypnotic cases were presented. Your readers, I know, are acquainted with the facts, but I confess it was the first time I had ever seen them developed, and as I saw two or three other phenomena developed which I had not before seen in print, I deem it of interest to narrate them.

The wand by which the magician—for it seemed like magic—developed the hypnotic state, was the little percussing hammer used for the detection of the tendon reflex. Holding the hammer like a pen in his hand, and passing two or three times in different directions over the face of the subject, the hypnotic state was developed. All the patients who were thus acted upon were previously afflicted with rigidity of one or more of their limbs. Immediately the hypnotic state manifested itself, the most complete flaccidity of the various members occurred, except those that were rigid before the hypnotic condition. A change, however, from the flaccid condition of the various members to that of rigidity, was produced immediately on rubbing the muscles which it was desired to contract. This contraction was very readily removed by gentle friction applied to the opposing muscles. When, however, the same influence was brought to bear on the limbs in a state of contraction before the hypnotism, it failed completely.

On lifting the eyelids so as to allow light to enter the eye during the hypnotic condition, a condition of catalepsy was immediately produced. This could be provoked unilaterally or bilaterally, at the will of the operator. The patient was awakened from sleep by simply blowing in the face.

I saw M. Dumontparlier produce similar effects in his wards. He set me to look at one of his patients in the face, and in less than half a minute she fell into the most complete hypnotic state. He put a bandage over her eyes, and another patient was observed to be in the same hypnotic condition. To this latter patient he applied, with a bit of cotton attached to a stick, a small quantity of ether to the dorsal part of the wrist. The arm flew to the most complete extension like a shot from a cannon. The same was applied to the other arm with a similar result.

This condition was relieved by blowing over the hand. Then a little ether—the patient being still in the hypnotic condition—was applied to the umbilicus, and immediately the most complete opisthotonus was developed. This condition was immediately relieved by blowing over the toes with a bellows.

Going to the former patient, who still had the bandage over the eyes, she manifested signs of wakefulness. M. Dumontpar lier rubbed her eyes a little, and pressed on a point in the left temple on an imaginary line connecting the occiput with the point of the nose, and she became unable to utter her own name or to give the name of any object that was presented to her. I you will allow me to mix up matters a little, I would like to add that I saw a few days ago, in the wards of M. Fournier, a case of syphilitic affection of the brain, in which the peculiar expression of countenance which the young man developed in trying to utter his own name—which he was utterly unable to do—forcibly reminded me of the expression of countenance of the partially hypnotic girl under the influence of pressure in the temple. The syphilitic patient nodded assent when his name or his age was given, with an expression of countenance exactly similar to the expression of the partially hypnotic girl.

This is not, as I said above, an account of any one individual clinic, so I will conclude by appending Charcot's table of

### TABETIC SYMPTOMS.

#### SPINAL.

*Sensitive.*—Lancinating pains; anæsthesia or hyperæsthesia (circumscribed); loss of the notion of position of limbs; sign of Romberg. *Muscular.*—Absence of reflex of knee; incoördination.

#### CEPHALIC.

*Sensitive.* — Lancinating pains; circumscribed anæsthesia or hyperæsthesia; gray atrophy of optic nerve; tinnitus aurium. *Muscular.*—Myosis spinal sign of Argyle Robertson; paresis of muscles of eye; diplopia; falling of the eyelids.

#### VISCERAL.

Disturbance of the functions of the stomach, intestines, retina, anus, bladder ; disturbance of the bladder; spasm of the larynx.

ATROPHIC.

Muscular atrophy; osseous lesions, especially in the joints (these lesions were illustrated by a large number of cases and pathological specimens); lesions of the skin; tona.

R. TILLEY.

---

### ARTICLE IX.

PARIS, July 31, 1882.

How different the surgical clinics in Paris are from those in Vienna—Professor Bilroth's clinic being taken as a sample of the latter. In Prof. Bilroth's clinic every assistant knows just what he has to do and it is done without noise or confusion, the operator seldom having to ask for anything. Carbolized water, about 2½ per cent., being in such abundance that the floor is often covered with it. In Paris the public clinics generally are marked by a great deal of confusion—exception being made to the clinic of Prof. Charcot. The larger eye clinics exhibit this confusion to perfection. The smaller eye clinics, however, are conducted with much greater order.

Electro-cautery is being used extensively here in various affections of the eye, I might almost say promiscuously. It is used in entropion, extropion, pannus, leucoma, ulcers of cornea, scleritis, anterior staphyloma, conical cornea, and detachment of the retina. It is exceedingly difficult, as every one knows, to ascertain the relative value to be attached to these operations in cases thus seen. The one advantage in many cases seems to be that it takes only a very little time to complete the operation, an advantage not always on the side of the patient. The operations on the lids, here in Paris, are not well done, and of all the thermo or electro-cautery operations it struck me that the operation for entropion of the lower lid was the most unsatisfactory; it leaves a most ugly wound for a long time after and the result must be very uncertain. I had the opportunity of demonstrating Dr. Hotz's operation for entropion in one of the best smaller clinics. The result was so satisfactory that on visiting the same clinic a week or so later I was shown two other cases in which the same operation had been performed, and the *Chef du Clinique* expressed himself greatly pleased with the operation. The only objection, which as you will see is no objection at all, is that it takes quite a little time to accomplish it thoroughly. I have spoken with several oculists here in Europe about this operation and have been astonished how few people who had read the description had properly understood and appreciated the main points.

In detachment of the retina M. De Wecker is using electrocautery two rows of points in the lower cul-de-sac—so, : : : : : : : ; whilst M. Abadie is thrusting a needle into the eyeball, attaching it to a battery and applying the other pole to the forehead, expecting in this to effect electrolysis. If this operation effects anything it certainly will not be by electrolysis; it would be absolutely impossible with a battery such as was used and the poles so far apart to effect such an operation.

Pilocarpine for detachment seems to have taken a back seat. I have seen pilocarpine, however, administered subcutaneously, to dispensary patients, and the patient sent into the waiting room to spit and sweat until he was tired and then walk home in the rain. I do not say this to recommend the practice, and I must say, in justice to the majority of the clinics, that I only saw it in one of the largest clinics. A good deal more attention, here in Paris, is given to the association of constitutional affections in affections of the eye than in Vienna. A good deal more attention is paid here to the field of vision. This is probably due to the researches of Charcot.

M. Paen is spoken of as one of the most successful surgeons in Paris. Consequently I availed myself of the opportunity of witnessing two of his ovariotomy operations. It is, of course, not necessary to describe the operation, but I will briefly pass in review such points as seemed to me noteworthy.

The chair or table is portable. A similar one he always takes with him for his operations in the country. Anæsthesia being produced, the part of the table at the foot is removed and two wire troughs are attached for the reception of the legs. The hands and feet are fastened to the table. The cover of the table being removable and divided down the middle, the patient is removed to bed with great ease. The tongue is drawn forward with a pair of forceps from the commencement of the operation. No carbolic acid is used. In place of it a solution of the peroxide of hydrogen in water is used as a spray. The apparatus for the spray is placed at about four feet from the patient, and the peroxide of hydrogen solution is supposed to contain six volumes of oxygen to one of water. His special direction is that this solution should be absolutely neutral. It is difficult to see how this can operate as an antiseptic, but I should observe that the same preparation is being used in several eye clinics.

The preliminaries of shaving, washing and covering the abdomen being accomplished, a long bold incision is made and an immediate and abundant use of hæmostatic forceps is exhibited. In the two operations I saw there was scarcely enough blood spilt to tinge the pail of water. As soon as the abdomen is open the wound is carefully protected by his assistants with clean soft napkins. He uses sponges, of course, but uses a good many more *soft clean napkins.* One of the assistant sisters always stood by a pile of about two dozen and ready to hand them to him at a word. A napkin, very soft, was even put into the abdomen instead of the sponge. No napkin was used twice for anything. There was nothing particularly noteworthy about the tying the pedicle. After the section, however, even part of the abdominal stump was taken up by the hæmostatic forceps previous to the final ligature. The ligatures were of silver. In stitching the abdomen the stitch was always taken from the inside. There was nothing peculiar or superior about the bandaging.

I paid a visit to the manufacturer of the peroxide of hydrogen in order to inquire something about the condition of acidity on which M. Paen seemed to place so much importance. He assured me that it was absolutely impossible to keep peroxide of hydrogen unless the solution was slightly acid. The difficulty being that on the slightest elevation of temperature the corks would fly and the bottles burst. I have since had some experience of that, for I bought of him a small quantity and requested of him to have the cork tied in. It was not tied in, however, and the consequence was my peroxide of hydrogen was spilt. The manufacturer here makes it at what he calls sixteen volumes; or one volume of the solution, if the quantity of peroxide of hydrogen contained in it were dissolved, would yield sixteen volumes or equal parts of oxygen. He acknowledged that it was not certain, but he would guarantee it to contain twelve volumes.

Professor Hayem, of Hôpital San Antoine, has been using it for some time past for the preservation of diabetic urine, which he makes use of as a diluting fluid for counting the blood corpuscles.

A system of what is called " Gavage " is being practiced here by M. Dujardin Beaumetz. This is practiced principally for consumptive patients who have no appetite. It consists in passing food into the stomach by means of a short œsophageal tube. The food consists of a thick fluid composed of meat specially prepared, dried and stirred up with milk. The preparation of the meat consists in cutting away as much as possible of the cellular tissue, warming it in water hot enough to cause the fat to rise to the top and then drying it at a temperature of 45 centigrade. The patients are said to gain weight and improve greatly.

A patient of considerable interest of late in Paris is a young woman at Hôpital Beaugon. Until lately she had been in a condition of absolute unconsciousness extending over a period of seventy-four days. During this period she gave birth to a four months' fœtus without any apparent effort and without any modification of the lethargic condition. She was first found in the street unconscious. At the suggestion of Prof. Charcot, cold douches were administered and restored consciousness. Electricity and various other expedients had previously been tried without effect.

R. TILLEY.

---

THE ideas of suicide originate in the circonvolutions of the brain, resulting from lesions in the frontal and parietal convolutions. So says Dr. Auguste Voisin.

